# Glioblastoma: Clinical Presentation, Multidisciplinary Management, and Long-Term Outcomes

**DOI:** 10.3390/cancers17010146

**Published:** 2025-01-05

**Authors:** David Sipos, Bence L. Raposa, Omar Freihat, Mihály Simon, Nejc Mekis, Patrizia Cornacchione, Árpád Kovács

**Affiliations:** 1 Department of Medical Imaging, Faculty of Health Sciences, University of Pécs, 7621 Pécs, Hungary; kovacsarpad@med.unideb.hu; 2 Dr. József Baka Diagnostic, Radiation Oncology, Research and Teaching Center, “Moritz Kaposi” Teaching Hospital, Guba Sándor Street 40, 7400 Kaposvár, Hungary; 3 Institute of Pedagogy of Health and Nursing Sciences, Faculty of Health Sciences, University of Pécs, Vörösmarty Str. 4, 7621 Pécs, Hungary; raposa.laszlo@pte.hu; 4 Department of Public Health, College of Health Science, Abu Dhabi University, Abu Dhabi P.O. Box 59911, United Arab Emirates; omar.mfreihat@adu.ac.ae; 5 Department of Oncoradiology, Faculty of Medicine, University of Debrecen, 4032 Debrecen, Hungary; simonm@med.unideb.hu; 6 Medical Imaging and Radiotherapy Department, University of Ljubljana, Zdravstvena Pot 5, 100 Ljubljana, Slovenia; nejc.mekis@zf.uni-lj.si; 7 Dipartimento di Diagnostica per Immagini e Radioterapia Oncologica, Fondazione Policlinico Universitario A. Gemelli IRCCS, 00168 Rome, Italy; patrizia.cornacchione@policlinicogemelli.it

**Keywords:** glioblastoma, prognosis, treatment, survival, outcome

## Abstract

Glioblastoma, the most common and aggressive primary brain tumor in adults, presents a formidable challenge due to its rapid progression, treatment resistance, and poor survival outcomes. Standard care typically involves maximal safe surgical resection, followed by fractionated external beam radiation therapy and concurrent temozolomide chemotherapy. Despite these interventions, median survival remains approximately 12–15 months, with a five-year survival rate below 10%. Prognosis is influenced by factors such as patient age, molecular characteristics, and the extent of resection. Patients with IDH-mutant tumors or methylated MGMT promoters generally have improved survival, while recurrent glioblastoma is associated with a median survival of only six months, as therapies in these cases are often palliative. Innovative treatments, including TTFields, add incremental survival benefits, extending median survival to around 20.9 months for eligible patients. Symptom management—addressing seizures, headaches, and neurological deficits—alongside psychological support for patients and caregivers is essential to enhance quality of life. Emerging targeted therapies and immunotherapies, though still limited in efficacy, show promise as part of an evolving treatment landscape. Continued research and clinical trials remain crucial to developing more effective treatments. This multidisciplinary approach, incorporating diagnostics, personalized therapy, and supportive care, aims to improve outcomes and provides a hopeful foundation for advancing glioblastoma management.

## Simple Summary

Glioblastoma is the most aggressive brain cancer and has challenging survival outcomes despite comprehensive treatment strategies. Typical survival for newly diagnosed patients is 12–15 months with a two-year survival rate below 30%. Treatment generally includes surgical resection, radiation, and temozolomide chemotherapy. Key factors impacting prognosis include patient age, molecular markers, and the extent of tumor resection. Younger patients and those with IDH-mutant or MGMT-methylated tumors often fare better. For recurrent cases, median survival drops to about six months, with treatments offering mostly palliative benefits. Innovations like tumor-treating fields (TTFields) provide a modest survival extension. Comprehensive care also requires managing symptoms like seizures and headaches, along with psychological support for patients and caregivers to improve quality of life. Although current therapies have limited efficacy due to glioblastoma’s resistance, ongoing clinical trials and research into targeted and immunotherapies provide hope for improved outcomes in the future.

## 1. Introduction

Glioblastoma, also known as glioblastoma multiforme (GBM), is the most aggressive and malignant form of primary brain tumor originating from astrocytes, the star-shaped glial cells that support nerve cells in the central nervous system. Classified as a grade IV astrocytoma by the World Health Organization, GBM is characterized by rapid proliferation, diffuse infiltration into surrounding brain tissue, and a high degree of genetic heterogeneity. This heterogeneity contributes to the tumor’s resistance to conventional therapies such as surgery, radiation, and chemotherapy [1]. Despite multimodal treatment approaches, including the use of temozolomide and radiotherapy, the median survival for patients diagnosed with glioblastoma remains approximately 12–15 months [2]. GBM’s complex biology, including dysregulated signaling pathways like the EGFR and PDGFRA, as well as resistance mechanisms such as the presence of glioma stem cells, underscores the challenge in finding effective treatments. Research is ongoing to explore novel therapeutic strategies, including targeted therapies, immunotherapies, and personalized medicine approaches, to improve patient outcomes [1,2,3].

Glioblastoma is the most common and lethal primary malignant brain tumor in adults, accounting for approximately 45–50% of all primary brain cancers. Its annual incidence is estimated at 3–5 cases per 100,000 individuals globally, with slightly higher rates observed in developed countries [4]. The peak age of diagnosis typically occurs between 65 and 75 years, and the disease shows a slight male predominance, with men about 1.5 times more likely to develop glioblastoma than women. Although glioblastoma can occur in all racial and ethnic groups, studies have shown that people of Caucasian descent tend to have a higher incidence compared to other populations [5]. The exact cause of glioblastoma is still unclear, but several risk factors have been identified. These include genetic predisposition (e.g., mutations in tumor suppressor genes like TP53), exposure to ionizing radiation, and certain hereditary syndromes such as Li–Fraumeni syndrome and neurofibromatosis type 1. However, lifestyle-related risk factors like smoking or diet have not been definitively linked to glioblastoma. The disease’s high incidence in older populations and poor prognosis highlight the need for further research into its underlying causes and potential preventive measures [6].

The pathophysiology of glioblastoma is marked by its aggressive growth, diffuse infiltration into surrounding brain tissue, and significant genetic and molecular heterogeneity. A key feature of glioblastoma biology is the dysregulation of signaling pathways that control cell proliferation, survival, and angiogenesis. Common molecular alterations include mutations in genes like the EGFR (epidermal growth factor receptor), TP53 (tumor protein p53), and PTEN (phosphatase and tensin homolog), all of which contribute to unchecked tumor growth and resistance to cell death [7,8].

One important molecular classification of glioblastomas is based on the presence or absence of isocitrate dehydrogenase (IDH) mutations. IDH-wildtype glioblastomas, which represent the majority of cases, are more aggressive and have a poorer prognosis compared to IDH-mutant glioblastomas, which are typically seen in younger patients and associated with better outcomes [9].

Another critical molecular feature is the methylation status of the O6-methylguanine-DNA methyltransferase (MGMT) promoter. MGMT is a DNA repair enzyme that counteracts the effects of alkylating chemotherapeutic agents like temozolomide. Methylation of the MGMT promoter silences this gene, reducing its repair activity and thereby increasing the tumor’s sensitivity to chemotherapy. Patients with MGMT promoter methylation typically have a more favorable response to treatment and better overall survival compared to those without it [10].

Glioblastoma typically presents with a range of neurological signs and symptoms, driven by the tumor’s location, size, and rate of growth within the brain. Common symptoms include persistent headaches, often characterized by a pattern of worsening pain due to increased intracranial pressure [11]. Patients frequently experience seizures, which may vary in type and intensity depending on the tumor’s location. Cognitive and behavioral changes, such as memory impairment, personality shifts, and confusion, are also prevalent, reflecting the tumor’s impact on various brain regions involved in higher-order processing [12]. Focal neurological deficits, including weakness or numbness in one part of the body, vision changes, and difficulties with speech or language (aphasia), are common when glioblastomas affect motor, sensory, or speech areas, respectively. As the disease progresses, symptoms typically worsen due to tumor growth and increased cerebral edema, often requiring urgent medical intervention to relieve intracranial pressure. The non-specific nature of many symptoms can lead to delays in diagnosis, underscoring the need for heightened awareness and prompt neuroimaging when new or worsening neurological symptoms are observed in adults [11,12,13].

## 2. Diagnosis of Glioblastoma Multiforme

The diagnostic approach for glioblastoma relies on a combination of advanced imaging, histopathological examination, and molecular diagnostic techniques to accurately identify and characterize the tumor. Magnetic resonance imaging (MRI) is the primary imaging modality due to its superior resolution and ability to differentiate soft tissues. Typical MRI features of glioblastoma include a contrast-enhancing mass with irregular borders, central necrosis, and surrounding vasogenic edema, often described as a “ring-enhancing” lesion on T1-weighted imaging. Advanced MRI techniques, such as diffusion-weighted imaging (DWI) and perfusion MRI, provide additional insights by assessing cellular density and vascularity, respectively, which are frequently elevated in glioblastomas due to their aggressive and infiltrative nature. Computed tomography (CT) is sometimes used, particularly in emergency settings, to identify mass effects or hydrocephalus, though it is generally less sensitive than MRI for glioblastoma [14,15].

Positron emission tomography (PET) imaging, particularly with amino acid tracers, has emerged as a valuable adjunct in the diagnostic and management approach for glioblastoma. Unlike conventional MRI and CT, which primarily assess anatomical and structural details, PET imaging provides functional insights by detecting areas of altered metabolism, which can be indicative of tumor presence and activity. For glioblastoma, ^18^F-fluorodeoxyglucose (FDG) PET has been used historically; however, it has limitations due to the high background uptake in normal brain tissue, which can obscure the tumor signal. Consequently, amino acid PET tracers, such as ^18^F-fluoroethyl-L-tyrosine (FET), ^11^C-methionine (MET), and ^18^F-DOPA, have proven more effective in distinguishing glioblastoma tissue from the surrounding healthy brain, due to the low physiological uptake of these tracers in normal brain tissue and their selective uptake in tumor cells [16,17].

Amino acid PET imaging enhances diagnostic precision by identifying tumor boundaries more accurately, which is especially valuable in cases where glioblastoma infiltrates surrounding brain regions. This functional imaging modality is also used to differentiate between tumor recurrence and post-treatment effects, such as radiation necrosis, which can present with similar enhancement patterns on MRI. Furthermore, amino acid PET imaging supports treatment planning by providing precise data on tumor metabolism and proliferation that can guide the delineation of target areas for surgery or radiotherapy [17].

In preoperative assessment, amino acid PET can help evaluate tumor infiltration into eloquent brain areas, aiding in surgical planning to maximize tumor resection while preserving critical functions. It also offers prognostic value, as higher tracer uptake in PET imaging has been associated with more aggressive tumor phenotypes and poorer outcomes. By integrating PET imaging—particularly with amino acid tracers—into the diagnostic pathway, clinicians can gain a more comprehensive understanding of glioblastoma’s metabolic characteristics, which complements structural imaging from MRI and CT, thereby enhancing the overall accuracy of diagnosis, treatment planning, and monitoring [14,15,16,17].

Definitive diagnosis requires the histopathological examination of tumor tissue, typically obtained through stereotactic biopsy or surgical resection. Under the microscope, glioblastoma is characterized by marked cellular atypia, high mitotic activity, microvascular proliferation, and areas of necrosis with surrounding “pseudopalisading” cells—a hallmark of this malignancy. Immunohistochemical staining can further confirm the glial origin of the tumor, often through markers like glial fibrillary acidic protein (GFAP) [18].

In recent years, molecular diagnosis has become an essential component of glioblastoma classification, with key molecular markers helping to inform prognosis and therapeutic approaches. For instance, the presence or absence of IDH mutations stratifies glioblastomas into IDH-mutant or IDH-wildtype subtypes, with the latter associated with a worse prognosis [9]. Another important molecular marker is the methylation status of the MGMT promoter, which, when methylated, indicates a greater likelihood of response to alkylating chemotherapy agents like temozolomide. Additional genetic alterations, including amplification of the EGFR gene and the loss of heterozygosity on chromosome 10q (often involving the PTEN tumor suppressor gene), further contribute to the molecular profile of the tumor and may inform targeted therapeutic options in the context of clinical trials [10]. This multimodal diagnostic approach combining imaging, histopathology, and molecular testing is critical to achieving an accurate glioblastoma diagnosis and developing an individualized treatment strategy aimed at improving patient outcomes [9,10,11,12,13,14,15,16,17,18].

## 3. Prognostic Factors and Staging

### 3.1. WHO Classification of Gliomas

The World Health Organization (WHO) classification of gliomas provides a standardized framework for diagnosing and grading gliomas based on histopathological and molecular characteristics. This classification system, recently revised in 2021, divides gliomas into several types and grades, primarily determined by the tumor’s cell of origin (e.g., astrocytomas, oligodendrogliomas) and specific genetic mutations that influence prognosis and therapeutic response [19]. A key update in the recent classification is the emphasis on molecular markers such as isocitrate dehydrogenase (IDH) mutations and 1p/19q codeletions, which help distinguish between astrocytomas and oligodendrogliomas. Gliomas are graded on a scale from I to IV, reflecting their aggressiveness and malignancy potential. Low-grade gliomas (grade I and II) generally exhibit slower growth and better prognosis, while high-grade gliomas (grade III and IV), including glioblastomas (grade IV astrocytomas), are more aggressive and invasive. The classification also acknowledges IDH-wildtype astrocytomas, which are molecularly similar to glioblastomas and carry a poor prognosis. By incorporating molecular markers alongside traditional histology, the WHO classification enhances diagnostic accuracy, guides treatment decisions, and provides a more consistent prognostic outlook for patients across clinical settings [19,20].

### 3.2. Staging, Grading, and Other Prognostic Markers

In gliomas, staging is not traditionally used as in other cancers due to their confinement to the central nervous system; instead, grading and molecular markers play crucial roles in assessing prognosis and guiding treatment. The World Health Organization (WHO) grading system for gliomas categorizes tumors from grade I to IV based on histopathological features like cellular atypia, mitotic activity, microvascular proliferation, and necrosis. Low-grade gliomas (grades I and II) typically exhibit fewer mitoses and slower growth, while high-grade gliomas (grades III and IV) are characterized by aggressive cellular proliferation and extensive infiltration into surrounding brain tissue, leading to poorer outcomes [19,20]. In addition to grading, molecular markers provide valuable prognostic information. Key markers include the isocitrate dehydrogenase (IDH) mutation, which is associated with better prognosis and is commonly found in lower-grade gliomas. Similarly, 1p/19q codeletion, typically observed in oligodendrogliomas, also correlates with a favorable response to chemotherapy and longer survival [20]. The methylation status of the MGMT (O6-methylguanine-DNA methyltransferase) promoter is another critical marker; methylation indicates increased sensitivity to alkylating agents like temozolomide and improved patient outcomes [10]. The WHO classification further distinguishes IDH-wildtype gliomas, which generally behave more aggressively and exhibit poorer prognosis than their IDH-mutant counterparts [19]. By integrating tumor grades with molecular and genetic markers, clinicians can more accurately predict disease progression, tailor treatment strategies, and provide patients with a clearer outlook on their expected prognosis [18,19,20].

## 4. Standard Treatment Options

### 4.1. Role of Resection, Craniotomy, and Surgical Approaches

Surgical resection plays a pivotal role in the management of glioblastoma, with the primary goal of reducing tumor burden to prolong survival and alleviate symptoms. The extent of resection (EOR) is a significant prognostic factor, as studies consistently show that achieving a maximal, or near-total, resection correlates with improved survival and delayed tumor progression [21]. During resection, neurosurgeons aim to remove as much tumor tissue as possible while preserving essential brain functions, necessitating precise preoperative planning and intraoperative guidance [22]. Craniotomy, the surgical opening of the skull, provides direct access to the tumor and can be tailored to the tumor’s location and extent. Advanced imaging techniques, such as functional MRI and diffusion tensor imaging (DTI), are often utilized preoperatively to map critical brain areas and white matter tracts, helping surgeons navigate around eloquent brain regions and minimize neurological deficits [23].

Intraoperatively, techniques such as neuronavigation, intraoperative MRI, and fluorescence-guided surgery using 5-aminolevulinic acid (5-ALA) enhance the visualization of tumor margins, enabling more precise resection. Awake craniotomy, a specialized approach used when the tumor is near functional areas, allows real-time monitoring of the patient’s speech, motor, and sensory functions, thus optimizing the EOR while preserving quality of life [22,23,24]. While complete resection is challenging due to glioblastoma’s diffuse and infiltrative nature, achieving maximal safe resection is critical for optimizing patient outcomes and enhancing the efficacy of adjuvant therapies like radiotherapy and chemotherapy. Postoperative imaging is typically performed within 24–48 h to assess the EOR, and any residual tumor volume helps guide subsequent treatment. The role of surgery in glioblastoma management, therefore, extends beyond simple tumor removal to encompass a strategic approach that combines advanced imaging, surgical precision, and adjunct therapies to offer patients the best possible prognosis and quality of life [23,24,25].

### 4.2. Radiation Therapy: Fractionated External Beam Radiation and Stereotactic Radiosurgery

Radiation therapy is a cornerstone in the management of glioblastoma, aiming to target residual tumor cells post-surgery to slow tumor progression and extend survival. The standard approach, fractionated external beam radiation therapy (EBRT), involves delivering controlled doses of radiation over multiple sessions, typically over a six-week course, with doses fractionated into daily sessions of around 2 Gy [26]. Fractionation allows healthy brain tissue time to repair between doses, minimizing side effects while maximizing damage to glioblastoma cells, which are less efficient at repairing DNA damage. EBRT has been shown to improve survival when used in conjunction with surgical resection and chemotherapy, often in a multimodal treatment regimen. Standard EBRT, especially when combined with concurrent temozolomide chemotherapy, is the current gold standard for newly diagnosed glioblastoma patients and has been shown to provide a median survival benefit [27].

Stereotactic radiosurgery (SRS) represents a more focused form of radiation therapy, delivering a high dose of radiation to a precise tumor area in a single session or a few sessions. SRS, often referred to by its common forms such as Gamma Knife or CyberKnife, is particularly suited for small, well-defined glioma recurrences rather than the initial treatment due to glioblastoma’s infiltrative growth patterns [28]. By concentrating radiation on a specific target, SRS minimizes exposure to surrounding brain tissues, reducing the risk of side effects. In recurrent glioblastoma cases, SRS can be an effective option to target isolated tumor remnants, providing palliative benefits and improving symptom control in selected patients. Both EBRT and SRS have limitations, primarily due to glioblastoma’s diffuse infiltrative nature, which often leaves microscopic disease beyond the irradiated field. However, combining radiation therapy with other treatments, such as chemotherapy or novel targeted agents, has shown promise in overcoming these challenges. Thus, radiation therapy, through EBRT and SRS, remains integral to glioblastoma management, carefully balanced to maximize tumor control while preserving neurological function [26,27,28,29].

## 5. Pharmacological Therapies

### 5.1. Chemotherapy: Temozolomide and Bevacizumab

Chemotherapy is a key component of glioblastoma treatment, with temozolomide (TMZ) as the standard chemotherapeutic agent due to its proven efficacy in improving survival when used concurrently with radiation therapy and as maintenance therapy thereafter. Temozolomide, an oral alkylating agent, works by methylating DNA at the O6 position of guanine, thereby inducing DNA damage that the tumor cells are unable to repair, ultimately leading to cell death [30]. The effectiveness of TMZ is often enhanced in patients with a methylated MGMT (O6-methylguanine-DNA methyltransferase) promoter, as this gene’s methylation reduces the tumor cells’ ability to repair TMZ-induced DNA damage [31]. For glioblastoma patients with MGMT promoter methylation, TMZ is especially beneficial and is associated with longer progression-free and overall survival. In cases where the MGMT promoter is unmethylated, alternative strategies or clinical trials may be considered, though TMZ remains a standard option due to its tolerability and moderate efficacy [32].

Bevacizumab, a monoclonal antibody targeting the vascular endothelial growth factor (VEGF), represents another therapeutic option, particularly in the recurrent glioblastoma setting. By inhibiting the VEGF, bevacizumab reduces tumor blood vessel formation (angiogenesis), thus limiting the nutrient and oxygen supply to the tumor and potentially slowing its growth [33]. Although bevacizumab has not demonstrated an improvement in overall survival in newly diagnosed glioblastoma, it has shown benefits in terms of symptom control and progression-free survival, particularly for patients with recurrent disease. Its use can lead to a reduction in peritumoral edema, often improving neurological symptoms and quality of life in selected patients [34]. However, due to the risks associated with bevacizumab, including hypertension, bleeding, and potential thromboembolism, its use is generally reserved for patients with symptomatic or radiographically progressive disease after initial therapies. Combining chemotherapy with other modalities, such as radiation and experimental targeted therapies, remains an area of active research aimed at overcoming glioblastoma’s resistance to conventional treatments. Thus, temozolomide and bevacizumab represent essential components in the therapeutic landscape for glioblastoma, each offering unique mechanisms and potential benefits based on the stage and progression of the disease [33,34,35].

### 5.2. Targeted Therapies and Immunotherapy Approaches

Targeted therapies and immunotherapy represent promising advancements in the treatment of glioblastoma, addressing the limitations of conventional therapies by aiming directly at molecular and immune vulnerabilities within the tumor [36]. Targeted therapies are designed to inhibit specific molecular pathways or mutations involved in glioblastoma growth and survival. Key targets include the epidermal growth factor receptor (EGFR), which is often amplified or mutated in glioblastoma, and phosphatidylinositol 3-kinase (PI3K)/Akt/mTOR pathways, critical for cell proliferation and survival. Agents such as tyrosine kinase inhibitors and mTOR inhibitors have been investigated; however, their efficacy has been limited in clinical settings, often due to the complex and heterogeneous nature of glioblastoma, as well as the blood–brain barrier, which restricts drug delivery [36,37]. Ongoing research is exploring combination strategies and new-generation targeted agents to overcome these challenges and improve clinical efficacy [38].

Immunotherapy, which aims to harness the body’s immune system to target and destroy cancer cells, has also emerged as a focal area of glioblastoma research. Immune checkpoint inhibitors, such as those targeting PD-1/PD-L1 pathways, have shown efficacy in several cancers but have encountered obstacles in glioblastoma, where the tumor microenvironment is highly immunosuppressive [39]. Despite this, personalized vaccines and cell-based therapies, such as dendritic cell vaccines and chimeric antigen receptor (CAR) T-cell therapies, are being developed to stimulate an immune response against specific glioblastoma antigens [39,40]. Dendritic cell vaccines prime the immune system by presenting tumor antigens, encouraging a T-cell response against glioblastoma cells. CAR T-cell therapy, on the other hand, involves engineering patients’ T-cells to target specific proteins on glioblastoma cells. Key targets for CAR T-cell therapy include EGFRvIII, a common mutation in glioblastoma, and IL13Rα2, an overexpressed receptor in glioblastoma that has been implicated in its aggressive behavior. Recent clinical trials with IL13Rα2-targeting CAR T-cells have shown promise, particularly in recurrent glioblastoma, demonstrating potential for tumor reduction in select cases [39,40,41].

The combination of immunotherapy with conventional treatments, such as radiation and chemotherapy, as well as the integration of immune-modulatory agents to overcome the immunosuppressive tumor environment, are areas of ongoing investigation. While targeted therapies and immunotherapy have yet to significantly impact glioblastoma outcomes on a broad scale, they provide a foundation for personalized approaches that may improve response rates and quality of life, marking a critical step forward in the pursuit of effective treatments for this aggressive cancer [36,37,38,39,40,41].

## 6. Emerging Therapies

### 6.1. Clinical Trials and Novel Treatments

Clinical trials and novel treatments are vital in advancing the management of glioblastoma, offering pathways to explore and validate new therapeutic options for this highly aggressive and treatment-resistant cancer. Clinical trials evaluate a wide range of innovative approaches, including refinements in conventional therapies, novel targeted therapies, immunotherapies, and combination regimens. These trials are essential for establishing the safety, efficacy, and optimal dosing of emerging treatments, providing data that can shape future standards of care. In early-phase trials, new compounds and treatment modalities, such as kinase inhibitors, oncolytic viruses, and epigenetic modifiers, are tested to determine tolerability and initial effectiveness, while later-phase trials assess their impact on survival and quality of life compared to current therapies [42,43].

Novel treatments in clinical development include advanced immunotherapy approaches, such as personalized vaccines, CAR T-cell therapy targeting glioblastoma-specific antigens, and immune checkpoint inhibitors that aim to reverse the tumor’s immunosuppressive environment. Gene therapy and oncolytic virotherapy are also being explored to deliver cytotoxic agents directly to tumor cells or stimulate immune responses within the tumor microenvironment [43,44]. Furthermore, precision medicine trials are investigating individualized treatment regimens based on the patient’s specific molecular tumor profile, with the goal of identifying targeted agents tailored to each patient’s genetic makeup [43,44,45].

Combination strategies are also a significant focus, integrating traditional treatments like temozolomide and radiation with novel agents to enhance therapeutic efficacy. Trials evaluating the combined use of targeted therapies, such as PI3K/mTOR inhibitors with radiation, or immune checkpoint inhibitors alongside chemotherapy, seek to overcome resistance mechanisms and increase tumor vulnerability [46]. Additionally, advanced imaging and biomarkers are used within trials to monitor treatment response and progression, enabling adaptive treatment strategies [47]. By rigorously testing and refining new therapies, clinical trials remain a cornerstone of glioblastoma research, paving the way for novel treatments that may improve survival and provide new hope for patients battling this challenging cancer [46,47].

### 6.2. Tumor-Treating Field (Optune)

Tumor-treating fields (TTFields), commercially known as Optune, represent an innovative and non-invasive therapeutic approach for glioblastoma that employs alternating electric fields to disrupt cancer cell division [48]. TTFields are delivered through a portable device with transducer arrays placed directly on the patient’s scalp, producing low-intensity, intermediate-frequency electric fields targeted to the tumor site. These fields interfere with cellular processes essential for tumor cell mitosis, particularly by disrupting microtubule formation and causing the abnormal alignment of intracellular organelles, which ultimately leads to cell cycle arrest and cancer cell apoptosis. TTField therapy has shown efficacy in extending progression-free and overall survival in newly diagnosed glioblastoma patients when used in combination with temozolomide, following standard surgery and radiation therapy [48,49,50].

The clinical benefit of TTFields was established through pivotal trials such as the EF-14 study [51], which demonstrated a significant improvement in survival rates for patients receiving TTFields alongside temozolomide compared to those receiving temozolomide alone. This technology is associated with a generally favorable safety profile, as its local delivery mechanism minimizes systemic side effects, with skin irritation at the device site as the most common adverse effect. However, concerns about the therapy are not uncommon. In clinical practice, some patients may refuse TTField treatment due to perceived or actual burdens associated with its use. Adverse events such as persistent skin irritation, discomfort from prolonged use of the device, and issues related to maintaining adherence for at least 18 h daily can pose significant challenges. Additionally, the visible nature of the device may contribute to psychological and social distress for some patients, impacting their willingness to use the treatment.

Research into TTFields is ongoing, with studies exploring its efficacy in combination with other treatments, such as targeted therapies and immunotherapies, to potentiate anti-tumor effects. Additionally, efforts are being made to refine the technology and optimize field intensities for better outcomes. TTFields represents a promising addition to the glioblastoma treatment landscape, offering a novel, mechanism-based approach that complements traditional therapies and provides new options for improving survival in patients facing this challenging diagnosis [48,49,50,51,52].

All aspects of comprehensive diagnostic approaches for glioblastoma, including imaging techniques, histopathological analysis, and molecular profiling, are summarized in Table 1.

## 7. Survival Rates and Quality of Life

### 7.1. Typical Survival Outcomes (Median Survival Rates)

Glioblastoma, despite advances in multimodal treatment strategies, remains associated with challenging survival outcomes due to its aggressive and infiltrative nature. Median survival rates for patients with newly diagnosed glioblastoma are approximately 12–15 months following standard treatment, which typically includes maximal safe surgical resection, radiation therapy, and concurrent chemotherapy with temozolomide. For those receiving the full regimen, including adjuvant temozolomide, survival rates at two years remain below 30%, with a five-year survival rate of less than 10%, underscoring the disease’s poor prognosis [2,3,4,5].

Certain molecular markers can impact survival outcomes. Patients with tumors that are IDH-mutant generally have a better prognosis and longer median survival than those with IDH-wildtype glioblastomas, the latter being more aggressive [9]. Similarly, glioblastoma patients with a methylated MGMT promoter, which indicates higher sensitivity to temozolomide, often experience improved survival compared to those without this marker. For recurrent glioblastoma, the prognosis remains particularly grim, with median survival typically around six months, as most therapies provide palliative benefit rather than curative potential in recurrent settings [10].

Innovative approaches, such as TTFields, have shown a modest improvement in survival when added to standard therapies, extending median survival to around 20.9 months for eligible patients. Ongoing clinical trials are exploring additional therapies aimed at improving these outcomes, but the overall survival rates for glioblastoma patients reflect the need for continued research and innovation in treatment options to address the disease’s resistance and rapid progression [50,51,52].

### 7.2. Factors Influencing Prognosis (Age, Genetic Markers, and Extent of Resection)

Prognosis in glioblastoma is influenced by a variety of factors, including patient age, genetic markers, and the extent of surgical resection, all of which play significant roles in determining survival outcomes and treatment response. Age is a critical prognostic factor; younger patients, especially those under 50, tend to have better survival rates than older individuals, likely due to a combination of improved physiological resilience and a higher likelihood of tolerating aggressive treatment. In contrast, older patients often present with more aggressive tumor subtypes and comorbidities that limit therapeutic options, contributing to a poorer prognosis [53,54].

Molecular and genetic markers further refine glioblastoma prognosis, with IDH mutation status being a particularly important predictor. IDH-mutant glioblastomas, though less common, generally have a more favorable prognosis and respond better to standard treatments compared to IDH-wildtype tumors, which are associated with a more aggressive clinical course. Another key marker is the methylation status of the MGMT (O6-methylguanine-DNA methyltransferase) promoter; methylated MGMT is linked to increased sensitivity to the alkylating agent temozolomide, which translates to improved survival and is commonly used as a predictive marker in treatment planning. Additionally, the presence of 1p/19q codeletion, typically found in oligodendrogliomas, is associated with a better prognosis and prolonged response to chemotherapy [9,10,50,51,52].

The extent of resection achieved during surgery is another major prognostic factor. Studies have shown that maximal safe resection, where as much tumor tissue as possible is removed, is associated with longer progression-free and overall survival. While complete resection is often limited by the infiltrative nature of glioblastoma, near-total or subtotal resections that significantly reduce tumor burden enhance the effectiveness of subsequent therapies, such as radiation and chemotherapy. Together, these factors—age, genetic profile, and the extent of resection—allow for a more personalized prognosis and are essential in guiding treatment strategies aimed at improving outcomes for glioblastoma patients [14,15,55].

All aspects of survival outcomes and prognostic factors in glioblastoma are comprehensively outlined in Table 2.

## 8. Palliative Care and Supporting Measures

### 8.1. Symptom Management (Seizures, Headaches, and Neurological Deficits)

Symptom management in glioblastoma is a crucial aspect of patient care, focusing on alleviating debilitating symptoms such as seizures, headaches, and neurological deficits that significantly impact quality of life. Seizures are a common symptom in glioblastoma, often occurring due to tumor-related cortical irritation [56]. Antiepileptic drugs (AEDs) are typically used for seizure management, with medications such as levetiracetam, valproate, and lamotrigine preferred for their efficacy and relatively low risk of drug interactions. Prophylactic AED use in patients without a history of seizures is generally not recommended, but close monitoring is essential given the high risk of seizure onset as the tumor progresses [56,57].

Headaches are another frequent symptom, often resulting from increased intracranial pressure due to tumor growth, cerebral edema, or obstructed cerebrospinal fluid flow. Corticosteroids, particularly dexamethasone, are commonly used to reduce peritumoral edema and alleviate headache symptoms, improving neurological function in many patients [58]. However, long-term corticosteroid use can lead to side effects such as muscle weakness, hyperglycemia, and osteoporosis, necessitating careful dosing and monitoring. In cases where corticosteroids are insufficient, other measures, such as analgesics or ventriculoperitoneal shunt placement for hydrocephalus, may be considered [59].

Neurological deficits, including weakness, sensory disturbances, and speech difficulties, depend on the tumor’s location and impact on surrounding brain tissue. Symptom management may involve physical, occupational, and speech therapy to support rehabilitation and maintain functional independence [60]. Additionally, palliative care interventions can address complex symptoms and provide psychological support for both patients and caregivers, helping to manage the emotional and cognitive challenges associated with progressive neurological decline [61]. Comprehensive symptom management, combined with regular assessment, allows clinicians to address the diverse and evolving needs of glioblastoma patients, aiming to improve quality of life and functional capacity throughout the course of the disease [60,61].

### 8.2. Psychological Support for Patients and Caregivers

Psychological support is a vital component of care for glioblastoma patients and their caregivers, addressing the emotional, cognitive, and social challenges associated with this aggressive and life-limiting disease [62]. A diagnosis of glioblastoma often brings significant psychological distress due to its poor prognosis and the impact of symptoms on daily functioning, leading to anxiety, depression, and feelings of helplessness. Providing patients with timely psychological support, such as counseling and cognitive behavioral therapy, helps them cope with the emotional burden of the illness and fosters resilience [63]. Support groups and psychoeducation also offer valuable platforms for patients to connect with others facing similar challenges, reducing feelings of isolation and providing practical coping strategies [62].

Caregivers, who often bear substantial emotional and physical responsibilities, are at high risk for burnout, depression, and anxiety. Support for caregivers includes counseling services, caregiver support groups, and respite care options to alleviate stress and promote well-being. Education about glioblastoma’s progression, expected symptoms, and treatment effects can empower caregivers with the knowledge needed to assist patients while managing their own emotional health. Hospice and palliative care teams often provide holistic support, addressing the spiritual, psychological, and physical aspects of care, which can help both patients and families navigate the advanced stages of the disease with dignity and comfort [64,65].

Psychosocial interventions, tailored to the unique needs of glioblastoma patients and their families, have been shown to improve quality of life, adherence to treatment, and overall patient satisfaction. Encouraging open communication between patients, caregivers, and healthcare providers further enhances the support system, allowing for compassionate and coordinated care that addresses the psychosocial impacts of glioblastoma [62,63,64,65].

## 9. Discussion

The management of glioblastoma presents unique challenges, encompassing complex diagnostic, therapeutic, and supportive care strategies [1,6]. Given its aggressive nature, infiltrative growth patterns, and resistance to standard therapies, glioblastoma requires a multimodal approach that combines surgery, radiation, chemotherapy, and supportive care. Each component plays a critical role, with the primary aim of prolonging survival, improving quality of life, and addressing the multifaceted symptoms associated with the disease [23,25,52].

From a diagnostic perspective, advancements in imaging and molecular profiling have enhanced clinicians’ ability to characterize glioblastoma with higher precision. MRI, supplemented with functional imaging techniques such as PET with amino acid tracers, provides insights into tumor metabolism and boundaries, essential for surgical planning and treatment monitoring [8,15,16]. Histopathological analysis and molecular markers, including IDH mutation, MGMT promoter methylation, and EGFR amplification, allow for a more personalized approach to treatment and have become fundamental in predicting treatment responses. As molecular classification continues to evolve, integrating these biomarkers will be vital for refining prognostic predictions and tailoring therapeutic strategies [2,31,32].

The therapeutic landscape of glioblastoma remains complex [6,7,9,26]. Surgical resection is foundational, with evidence showing improved survival associated with maximal safe resection. Radiation therapy, specifically fractionated external beam radiation, and emerging technologies such as stereotactic radiosurgery (SRS) are integral in targeting residual disease post-surgery, although the efficacy of these modalities remains limited by the infiltrative nature of glioblastoma [26]. Chemotherapy with temozolomide has become a standard, particularly for patients with MGMT promoter methylation, which confers greater chemosensitivity. Targeted therapies, including inhibitors for pathways like EGFR and PI3K/mTOR, have shown some promise, though challenges persist due to tumor heterogeneity and resistance mechanisms. Immunotherapy, while effective in many cancers, faces barriers in glioblastoma due to its immunosuppressive tumor microenvironment. Nonetheless, experimental approaches like CAR T-cell therapy and vaccines are being actively explored, with early-phase trials providing hope for future treatment options [30,31,46,58].

Emerging treatments like tumor-treating fields (TTFields), notably Optune, introduce an innovative approach by utilizing alternating electric fields to disrupt tumor cell division, demonstrating modest survival benefits when combined with standard therapies. This non-invasive modality offers a unique mechanism of action that may complement conventional treatment, particularly in newly diagnosed patients, and underscores the importance of developing diverse strategies against glioblastoma’s resistance [46,49,51].

Supportive care, encompassing symptom management and psychological support, is paramount for maintaining quality of life in glioblastoma patients [62,63]. Symptom control measures, including antiepileptic drugs for seizure management, corticosteroids for edema-related headaches, and rehabilitative therapies for neurological deficits, are essential to mitigate the debilitating symptoms of glioblastoma. Psychological support for both patients and caregivers is equally important, given the profound emotional toll of the disease [64]. Structured support groups, counseling, and psychosocial interventions provide patients and their families with coping mechanisms, emotional resilience, and practical tools for navigating the disease’s progression [62,63,64].

## 10. Conclusions

Glioblastoma remains a significant challenge in neuro-oncology, requiring a multidisciplinary approach that combines advanced diagnostics, innovative therapies, and comprehensive supportive care. While current treatments like surgery, radiation, and chemotherapy have limitations, emerging strategies such as tumor-treating fields, targeted therapies, and immunotherapies show promise despite challenges posed by the tumor’s heterogeneity and immunosuppressive microenvironment. Holistic care, including symptom management and psychological support, is vital for improving patients’ quality of life and addressing the emotional toll on patients and caregivers.

Collectively, the comprehensive approach to glioblastoma underscores the need for continued research and innovation. Novel therapies, optimized diagnostic techniques, and tailored supportive care measures aim to address the gaps in current treatment options and enhance patient quality of life. With ongoing clinical trials and research into molecular and immunological therapies, there is hope for more effective strategies that may one day transform glioblastoma from a terminal diagnosis into a manageable condition, improving outcomes for patients and easing the burden on their caregivers. The integration of personalized medicine and supportive care within the therapeutic framework for glioblastoma represents a forward-looking approach that holds promise for the future of glioblastoma management.

## Figures and Tables

**Table 1:** Comprehensive diagnostic approaches for glioblastoma: imaging, histopathology, and molecular profiling.

Imaging Techniques	Magnetic Resonance Imaging (MRI)	- Standard MRI: T1-weighted, T2-weighted, and FLAIR Sequences - Contrast-Enhanced MRI - Advanced MRI Techniques: * Diffusion-Weighted Imaging (DWI) * Perfusion MRI * Magnetic Resonance Spectroscopy (MRS)
	Computed Tomography (CT)	- Role of CT in Emergency Settings - Limitations Compared to MRI
	Positron Emission Tomography (PET) Imaging	- Overview of PET in Brain Tumor Imaging - 18F-Fluorodeoxyglucose (FDG) PET - Amino Acid PET Tracers: * 18F-Fluoroethyl-L-Tyrosine (FET) * 11C-Methionine (MET) * 18F-DOPA
Histopathological Examination		- Biopsy and Surgical Resection - Histological Characteristics: * Cellular Atypia and High Mitotic Activity * Microvascular Proliferation and Necrosis * Pseudopalisading Necrosis
Molecular Diagnostics	Importance of Molecular Profiling in Glioblastoma	- Key Molecular Markers: * Isocitrate Dehydrogenase (IDH) Mutation Status * IDH-Wildtype vs. IDH-Mutant Subtypes * MGMT Promoter Methylation Status * EGFR Amplification and Mutations * PTEN Loss and Chromosome 10q Deletions
Differentiation from Other Conditions		- Distinguishing Glioblastoma from Other Brain Lesions - Tumor Recurrence vs. Radiation Necrosis - Use of Advanced Imaging and PET for Differential Diagnosis
Role of Functional Imaging in Preoperative Planning		- Mapping of Tumor Boundaries - Evaluation of Infiltration in Eloquent Brain Areas - Functional Data for Surgical and Radiotherapy Planning
Prognostic and Predictive Value of Diagnostic Findings		- Imaging and Molecular Markers as Prognostic Indicators - Impact of PET and Molecular Profiling on Patient Outcomes
Future Directions in Glioblastoma Diagnosis		- Emerging Imaging Techniques - Advancements in Molecular and Genetic Profiling - Potential Role of Liquid Biopsies and Biomarkers

**Table 2:** Survival outcomes and prognostic factors in glioblastoma.

Typical Survival Outcomes (Median Survival Rates)	Standard Treatment Survival Rates	Glioblastoma patients receiving standard treatments (surgical resection, radiation therapy, and temozolomide) have a median survival of 12–15 months. Two-year survival rates are below 30%, and five-year survival rates are less than 10%.
	Impact of Molecular Markers	Patients with IDH-mutant glioblastomas have a better prognosis compared to those with IDH-wildtype tumors, which are typically more aggressive. Similarly, patients with methylated MGMT promoters often show improved survival due to increased sensitivity to temozolomide.
	Recurrent Glioblastoma	For recurrent glioblastoma, prognosis is poor, with median survival around six months, as treatments are generally palliative rather than curative at this stage.
	Innovative Approaches (TTFields)	Tumor-treating fields (TTFields), when added to standard therapies, modestly improve survival, extending median survival to approximately 20.9 months in eligible patients.
Factors Influencing Prognosis		
Age		Age is a critical prognostic factor, with younger patients (especially under 50) generally experiencing better survival due to physiological resilience and tolerance for aggressive treatment. Older patients often present with aggressive tumors and comorbidities, resulting in a poorer prognosis.
Genetic Markers	IDH Mutation Status	IDH-mutant glioblastomas are associated with better survival outcomes than IDH-wildtype tumors, which exhibit a more aggressive clinical course.
	MGMT Promoter Methylation	Methylated MGMT promoter status correlates with higher sensitivity to temozolomide and improved survival, making it a valuable prognostic and predictive marker in treatment planning.
	1p/19q Codeletion	Found in oligodendrogliomas, 1p/19q codeletion is associated with a better prognosis and increased responsiveness to chemotherapy, contributing to prolonged survival.
Extent of Resection	Maximal Safe Resection	Maximal safe resection (removal of as much tumor tissue as possible) is associated with improved progression-free and overall survival. Near-total resections enhance the efficacy of radiation and chemotherapy by reducing tumor burden, even if complete resection is often unattainable due to glioblastoma’s diffuse nature.
